# Serum amyloid A—A potential therapeutic target for hyper-inflammatory syndrome associated with COVID-19

**DOI:** 10.3389/fmed.2023.1135695

**Published:** 2023-03-16

**Authors:** Eman M. Almusalami, Anthony Lockett, Albert Ferro, John Posner

**Affiliations:** ^1^Centre for Pharmaceutical Medicine Research, King’s College London, London, United Kingdom; ^2^School of Cardiovascular and Metabolic Medicine and Sciences, British Heart Foundation Centre for Research Excellence, King’s College London, London, United Kingdom

**Keywords:** serum amyloid A, target, hyperinflammation, COVID-19, inflammation, cytokines, arthritides

## Abstract

Serum amyloid-A (SAA) is associated with inflammatory disorders such as rheumatoid arthritis, Familial Mediterranean Fever, sarcoidosis, and vasculitis. There is accumulating evidence that SAA is a reliable biomarker for these autoinflammatory and rheumatic diseases and may contribute to their pathophysiology. Hyperinflammatory syndrome associated with COVID-19 is a complex interaction between infection and autoimmunity and elevation of SAA is strongly correlated with severity of the inflammation. In this review we highlight the involvement of SAA in these different inflammatory conditions, consider its potential role and discuss whether it could be a potential target for treatment of the hyperinflammatory state of COVID-19 with many potential advantages and fewer adverse effects. Additional studies linking SAA to the pathophysiology of COVID-19 hyper-inflammation and autoimmunity are needed to establish the causal relationship and the therapeutic potential of inhibitors of SAA activity.

## 1. Background

In 1961, Benditt and Eriksen identified a protein by gel electrophoresis present in cases of chronic or recurrent inflammatory conditions, characterized by protein deposition; they called this protein “amyloid of unknown origin” (1). Subsequently, Levin et al. (1972) presented the full amino acid sequence for the protein found in this secondary amyloidosis which was called amyloid-A (2). Antibodies were then raised against amyloid fibril protein from a patient with systemic amyloidosis. The antibodies were used to identify a component in serum antigenically related to the amyloid protein and it was concluded that this could be a precursor of the tissue fibril protein amyloid (3); it was named serum amyloid-A (SAA).

SAA was initially studied as fibrils that are deposited in secondary amyloidosis. However, SAA is also an acute phase reactant (APR) protein that circulates in very low concentrations (<10 mg/L) in normal conditions but which can increase up to 1,000-fold within 24 h in response to inflammation, infection, and autoimmune disease (4). In this review, we aimed to study the potential of SAA to be a therapeutic target to combat hyper-inflammatory syndrome associated with COVID-19 based on the common feature of inflammation between arthritides, vasculitis, and COVID-19 hyperinflammation.

## 2. Literature review

**Figure 1:**
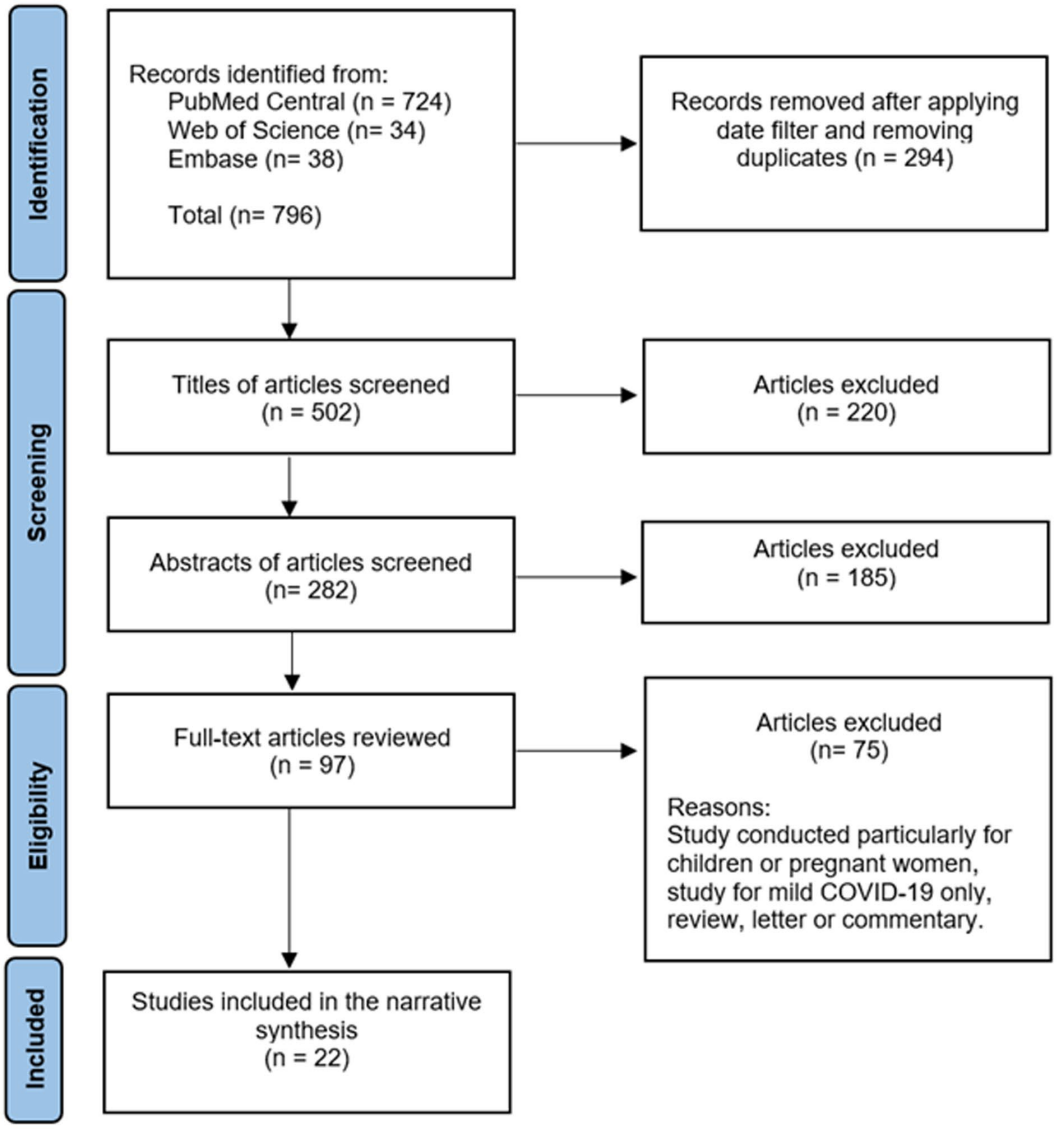
Flow chart demonstrating the literature review and the study selection process.

A comprehensive literature review was conducted using PubMed Central, Embase, and Web of Science databases to identify articles exploring the association of SAA with COVID-19. The following string was used: [(COVID-19 OR SARS-CoV-2 OR “coronavirus disease-2019” OR “covid 19” OR coronavirus OR “corona virus” OR “novel coronavirus” OR “coronavirus disease 2019” OR “coronavirus 2019” OR 2019-nCoV) AND (“Cytokine Release Syndrome” OR “Systemic Inflammatory Response Syndrome” OR “hyper inflammatory syndrome” OR “cytokine storm” OR “hyper inflammatory response” OR “hyperinflammatory response” OR “hyperinflammatory syndrome” OR inflammat*) AND (“Serum Amyloid A Protein” OR “serum amyloid A” OR “serum amyloid-A” OR “serum amyloid A peptide” OR SAA)]. Only full-text English language articles published in 2019 or after were included. Exclusion criteria were studies with children or pregnant women, studies without analysis of severe COVID-19, articles where only mild COVID-19 cases were analyzed, and study designs of letters, editorials, commentaries, reviews, opinions or perspectives. The initial literature search using the search string generated 796 articles from the three different databases. After removing duplicates and applying the filter for the date between 2019 to 2021 to ensure that only COVID-19 studies were included, 502 articles remained. After title and abstract screening, 97 articles were eligible for full-text articles assessment. Finally, 21 articles met the eligibility criteria. In addition, one article was found by a manual search in the reference lists of the retrieved articles. Therefore, 22 articles were included in this review. See Figure 1. Results were summarized in a narrative manner to address the association of SAA with COVID-19. Findings in this article not related to COVID-19 were analyzed with the literature review as they are general information regarding SAA, and not specifically related to COVID-19.

## 3. SAA association with cytokines and chemokines

SAA has role in inflammation since it has proinflammatory activities during acute inflammation through induction of pro-IL-1β synthesis, activation of NLRP3 (NOD-, LRR- and pyrin domain-containing protein-3) inflammasome, and induction of some cytokines and chemokines as an APR (5, 6). Moreover, SAA has chemotactic effect through stimulating the migration of many cells (6). Therefore, SAA is associated with inflammatory disorders. On top of that, SAA has an active role in keeping homeostasis during inflammatory process through preventing injury progression and repairing damaged tissues (6).

**Figure 2:**
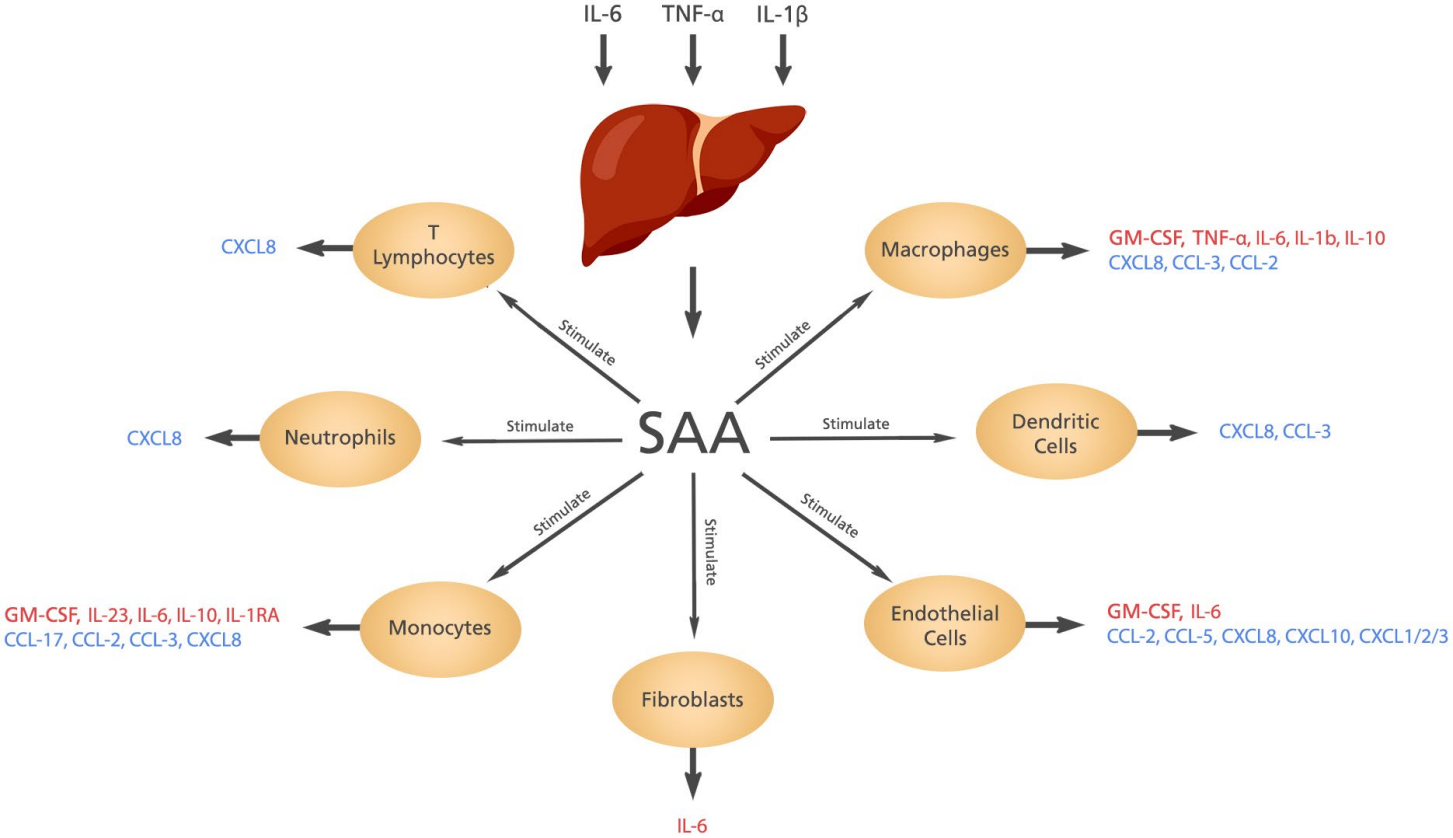
The cytokines and chemokines induced by SAA. Elevated levels of IL-6, IL-1β, and TNF-α stimulate the liver to produce SAA which in turn induces the production of different cytokines and chemokines from different cells.

SAA is synthesized in the liver in response to some cytokines including interleukin (IL)-6, tumor necrosis factor-alfa (TNF-α) and IL-1β. The contribution of SAA to inflammation includes induction of cytokines and chemokines, chemotactic activity, and activation of the NLRP3 inflammasome (5, 6). The cytokines and chemokines produced by SAA are shown in Figure 2.

## 4. SAA in inflammatory arthritides and vasculitis

There is accumulating evidence that SAA is a reliable biomarker for several autoinflammatory and rheumatic diseases, and that it is involved in the pathophysiology of rheumatoid arthritis (RA), familial Mediterranean fever (FMF), sarcoidosis, and vasculitis.

RA is characterized by progressive inflammation with different elevated cytokines; IL-1, TNF-α, IL-6, granulocyte-macrophage colony-stimulating factor (GM-CSF), macrophage colony-stimulating factor (M-CSF), transforming growth factor-beta (TGF-β), and chemokines; monocyte chemoattractant protein-1 (MCP-1), macrophage inhibitory protein-1 (MIP-1), and epithelial-neutrophil activating peptide (ENA) (7). Severe RA is characterized by widespread inflammation with systemic and extra-articular manifestations frequently involving the heart, lungs, blood vessels, and eyes (8). SAA is significantly elevated in the serum and the synovial fluid of RA patients with acute exacerbations compared to healthy controls; indicating the local production of SAA (9, 10). To further support this finding, all cytokines known to stimulate SAA synthesis, namely IL-6, IL-1β, and TNF-α, are elevated in patients with RA. High concentrations of IL-6 are presented in arthritic joints and blood, and together with IL-1β and TNF-α correlate with RA severity (11). SAA has been shown to be localized in both the lining and the sub-lining layer of synoviocytes and monocytes/macrophages of atherosclerotic lesions and some endothelial cells of patients with RA (12). Both formyl peptide receptor like-1 (FPRL-1) and the SR-B1, which are known SAA receptors, are expressed in synovial tissue (10, 12). Interestingly, Chambers et al. (1983) reported that SAA levels were increased while C-reactive protein (CRP) levels were normal in 40% of 185 patients with RA included in their study, but all patients with normal SAA levels had normal CRP levels even after adjusting for differences in demographics (13). These findings suggest that SAA is a more sensitive biomarker for RA compared to other inflammatory biomarkers such as CRP or erythrocyte sedimentation rate (ESR). Therefore, some studies recommended using SAA level to monitor RA disease activity in patients receiving TNF-α inhibitors. These drugs can reduce CRP without reduction in disease activity while SAA is strongly correlated with disease severity in patients receiving these drugs (14, 15). Moreover, it was shown that there is a significant correlation between serum SAA concentration and RA activity, and persistent elevation of SAA levels demonstrated subclinical inflammation in patients with RA (9, 16). It is worth noting that persistent elevation of SAA can lead to the development of amyloidosis since RA constitutes 12–21% of amyloidosis cases (17). The chemotactic activity of SAA was confirmed in the synovial fluid of RA patients with induction, migration, adhesion, and tissue infiltration of polymorphonuclear leukocytes and monocytes (18). Moreover, SAA can induce the production of cartilage degrading protease, matrix metalloproteinase-3 (MMP-3), which is consistent with the association between acute phase protein and progressive joint damage (12). SAA can induce cytokines and chemokines and in particular has been shown to potently induce IL-8 and MCP-1 chemokines in RA patients associated with aggravation of the disease (7).

FMF is a devastating genetic inflammatory condition characterized by elevation of several cytokines including Interleukin-1 receptor antagonist (IL-1Ra) and IL-1β (19, 20). SAA levels were found to be elevated in patients with FMF at diagnosis, during attacks as well as between attacks indicating the sustained subclinical inflammation (21, 22). One study showed that SAA levels were above the normal reference range in more than 95% of children with FMF in the attack free period even though half of the children had not experienced an attack within the last year; supporting the finding that SAA can be a biomarker for FMF subclinical inflammation (23). Moreover, SAA levels decreased significantly upon increasing the colchicine dose supporting the use of SAA level to guide colchicine therapy (23). While only 31% of the patients with FMF had an elevated CRP level, SAA concentrations were increased in 79% of the FMF patients (24).

Sarcoidosis is a multisystem inflammatory disease mainly affecting the lungs. Inflammation in sarcoidosis is associated with elevated levels of IL-1β, IL-6, TNF, and CCL2 (25). Looking to the pathophysiology of the sarcoidosis, SAA has a major role in regulating the inflammation through TLR-2. Bargagli et al. (2011) found that SAA was expressed in all sarcoidosis patients’ serum, but SAA was not expressed in the healthy controls (26). One study showed that SAA was expressed more in sarcoidosis granuloma than any other granulomatous diseases (27). Moreover, some studies reported that SAA level was significantly higher in patients with active sarcoidosis than patients with inactive sarcoidosis (26, 28, 29). However, there is conflicting evidence regarding the correlation of SAA levels with the disease severity (30, 31).

Vasculitis is a feature of many diseases; some of these are reviewed here, in particular their association with SAA. It was discovered that SAA has a role in the pathogenesis of the giant cell arteritis. Beside SAA properties of proangiogenesis, SAA has the ability to induce angiogenesis and cell growth mediated by the TLR-2 (31). Furthermore, one study showed that SAA is secreted in the inflammed temporal arteries (32). Hocevar et al. (2016) demonstrated that SAA levels were correlated with early relapse in patients having giant cell arteritis receiving corticosteroids (33). Therefore, SAA levels can be used for early identification of non-responders to corticosteroids. In addition, Dartevel et al. (2020) reported the clinical utility of SAA level in differentiating patients with active giant cell arteritis from patients with inactive disease (34). Interestingly, Van Sleen et al. (2019) found that SAA levels correlated significantly with serum IL-6 in patients with giant cell arteritis as SAA stimulates the IL-6 production (35).

Interestingly, of 12 biomarkers studied for Henoch-Schonlein purpura diagnosis, Purevdorj et al. (2018) found that SAA was the most sensitive biomarker (36). To support this finding, one study reported that SAA levels were significantly higher in the serum of patients with Henoch-Schonlein purpura than healthy controls; the average SAA levels were 12-fold higher (37). These two studies support the use of SAA levels as a biomarker in patients with Henoch-Schonlein purpura.

Two studies found that SAA levels were significantly higher in patients with active Takayasu arteritis than patients with inactive disease (38). Furthermore, SAA levels were higher in patients with inactive Takayasu arteritis than healthy controls (38). Additionally, Nair et al. (2017) demonstrated that SAA levels were aligned with the response to methotrexate, azathioprine, and mycophenolate mofetil during the follow up period; responders had significantly lower SAA levels than at baseline while there were no significant changes in the SAA levels for non-responders (39).

## 5. SAA in the hyperinflammatory syndrome associated with COVID-19

COVID-19 hyperinflammation is a complex interaction between infection and autoimmunity. Several reports showed that excessive immune response against SARS-CoV-2 breaks the natural self-tolerance maintained by the body’s immune system (40, 41). COVID-19 hyperinflammation pathology is caused by the immune system rather than direct viral virulence, and it is recognized that the immune response to the SARS-CoV-2 contributes to multiorgan failure if not treated appropriately (42). Consistent with this, clinical deterioration caused by hyperinflammation often occurs seven to 10 days after onset of symptoms when viral load is starting to decline (43, 44). Common features of the extensive systemic inflammation of COVID-19 include pneumonitis, vasculitis, and pericarditis.

Some studies showed that more than 83% of the COVID-19 patients had an abnormally elevated SAA level (45–47). Other studies reported significant elevation of SAA levels in COVID-19 patients compared with healthy control groups (48–50). In some studies, SAA levels were increased in COVID-19 patients even in mild/moderate disease, and the increment correlated with disease severity; significant difference in the SAA levels was reported between mild and more severe disease; *p* < 0.05 (45, 51–55).

Receiver Operator Characteristic (ROC) curve analysis in COVID-19 severity reported AUC values of 0.818, 0.835, and 0.947 in three different studies (52, 55, 56). In addition, Liu, Q. et al. (2020) showed that for each unit increase in SAA level, the risk of patients with non-severe COVID-19 developing severe disease increased significantly; OR = 4.212 and *p* = 0.039 (51). One study showed that a cut-off value for disease severity of 157.9 mg/L (56), and another showed that a SAA level greater than 100.02 mg/L can be used as a warning sign to closely observe the patient and possibly prevent COVID-19 progression (52).

Additionally, a cohort study with a sample size of 3,265 patients showed that SAA could serve as a biomarker for COVID-19 prognosis as SAA level > 12.4 mg/L showed increased risk of death using Kaplan–Meier survival curves (54). Several studies reported that SAA could serve as a biomarker for disease progression (45, 50, 53, 54, 57). Li, H. et al. (2020) showed that ROC curve of SAA in predicting COVID-19 progression had an AUC of 0.856 with a cut-off value of 159 mg/L (53).Surprisingly, Zhang, J. et al. (2020) showed no significant difference in the SAA level between severe and non-severe groups (47), but there were no critically ill patients included in that study. Moreover, there may be only a small difference in the SAA level between non-severe (specifically moderate patients) and severe patients as the study defined patients into severe and non-severe groups only.

Furthermore, two studies reported characteristics of death from COVID-19 showing that SAA levels were elevated (58, 59). Studying patients’ characteristics died of COVID-19 Yang et al. (2020) found that patients died of COVID-19 complications had higher SAA levels than patients died of COVID-19 without complications; *p* < 0.01 (59). The most common complication was ARDS (59).

Pieri et al. (2021) reported that SAA levels were higher in non-survivors than survivors from COVID-19; median SAA level in non-survivors was 740 mg/L versus 487.5 mg/L in survivors; *p* < 0.01 (60). On the other hand, Cheng et al. (2020) showed that SAA levels were not significantly lower in survivors than non-survivors before treatment (56). However, it was significant after treatment with glucocorticoids, antiviral, and nutritional support when secondary infection or worsening COVID-19 hyperinflammation have developed. The latter explanation is reasonable as the AUC and sensitivity for the ROC analysis for SAA level were the highest on day-7 after treatment compared to day-3 or day-5. In addition, receiving glucocorticoids is considered to be a confounder for SAA measurement. The study did not report the number of patients receiving glucocorticoids in each group nor was a sensitivity analysis performed to confirm the results even though the main aim of the study was to assess the prognostic value of SAA in COVID-19 patients.

Comparing SAA levels with other diseases, Bhatraju et al. (2021) performed a cohort study with a sample size of 171 ICU patients aiming to compare plasma biomarkers in sepsis between COVID-19 and non-COVID-19 patients. SAA concentrations were 2.9-fold greater in ICU COVID-19 patients than ICU non-COVID-19 patients (95% CI 1.67–5.28; *p* < 0.001) (61). Moreover, this study informed that SAA level was associated with ARDS in COVID-19; aRR = 1.27, supporting the findings of other studies.

Many studies showed that SAA levels correlated with mortality from COVID-19 with significantly more deaths in patients with high SAA levels (48, 51, 60).

## 6. Common features of inflammation associated with elevation of SAA

Some inflammatory arthitidies and COVID-19 hyperinflammation may lead to massive inflammation, inducing tissue destruction resulting in organ failure (24). Hyperinflammation associated with COVID-19 elicited an uncontrolled immune response by alveolar epithelial cells and T-cell activation in the lung, triggering excessive production of pro-inflammatory cytokines and attracting neutrophils and macrophages to the lung. This pathophysiology is a crucial feature of inflammatory arthritides including the barrier damage, activation of T-cells, cytokine production, neutrophils, and macrophages influx (24).

The hierarchical order of cytokines involved in COVID-19 hyperinflammation is not well-known yet (62). However, the contributing cytokines are the same as for most inflammatory arthritides, namely IL-6, IL-1β, and TNF-α. These cytokines are the current therapeutic targets for some of the inflammatory arthritides and autoimmune inflammatory diseases, and they seem to have a fundamental role in COVID-19 hyperinflammation (62). Many drugs indicated for inflammatory arthritides and autoimmune inflammatory diseases are now studied to be repurposed to treat COVID-19 hyperinflammation.

COVID-19 hyperinflammation results from a combination of defective or delayed first line defence followed by persistent hypercytokinemia (63, 64). Interferon is needed for proper T-cell activation to carry on immune functions to clear the virus. Undetectable or low levels of interferon in patients with severe and critical COVID-19 at the beginning of the disease probably contribute to the exaggerated response to infection and dysfunctional adaptive immune response (65, 66).

The concentration of SAA needed to induce cytokines is 1–10 mg/L or less (6). However, a higher concentration is needed for production of chemokines; 125 mg/L is needed for IL-8 stimulation from different cells (6, 67). However, a concentration of only 0.025 mg/L is needed for MCP-1 production. Meanwhile, the concentrations of SAA in severe COVID-19 patients exceed the SAA concentrations needed to induce cytokines and chemokines.

High SAA concentrations (*ca.* 50 mg/L) stimulate the oxidative burst from neutrophils (68). This response was reported in the COVID-19 hyperinflammation, leading to a cascade of events resulting in pathological host response (69). To support the pathological role of SAA with neutrophils in COVID-19 hyperinflammation, laboratory findings in patients with hyperinflammation showed high neutrophils:lymphocytes ratio (70). In addition, most patients with severe or critical COVID-19 had a SAA level of more than 50 mg/L demonstrating the ability of SAA to induce its pathological role in stimulating oxidative stress from neutrophils during hyperinflammation.

In COVID-19 hyperinflammation, NLRP3 inflammasome is activated, leading to synthesis of more cytokines and chemokines (64). Activating the NLRP3 inflammasome is one of the SAA functions through the P2X7 receptor and cathepsin B-sensitive pathway leading to IL-1β secretion from macrophages (71). Moreover, in a concentration as low as 10 mg/L of SAA, SAA has been reported to stimulate the migration of monocytes, neutrophils, endothelial cells, mast cells, T-cells, immature dendritic cells, and smooth muscle cells (6, 11). Investigating the possible reasons for lymphopenia associated with COVID-19 hyperinflammation, T-cell migration to the inflamed tissues is a possible mechanism that may be caused by SAA or other chemokines. The above summary supports the role of SAA in the pathophysiology of COVID-19 hyperinflammation.

## 7. Hypothesis: SAA as a target for treatment of inflammatory conditions

Elevation of SAA is clearly a feature of some inflammatory arthritides and the hyperinflammatory state of COVID-19. SAA can induce expression of IL-17 by T-helper (Th)17 subset of CD4^+^ cells, which play an important pathogenic role in several autoimmune conditions including RA, inflammatory bowel disease, psoriasis, and multiple sclerosis (72). Therefore, it is reasonable to hypothesise that SAA may be a major contributor to these inflammatory states and could be a potential therapeutic target.

The 3D-structure of SAA is known, allowing for prediction of potential binding sites. A synthetic 5-MER peptide with the amino acid sequence MTADV known as Amilo-5MER, has been shown to bind to SAA, interfere with SAA assembly and cytokine release. Its anti-inflammatory activity has been demonstrated in mouse models of RA, IBD and multiple sclerosis, namely collagen induced arthritis, 2,4,6-trinitrobenzenesulfonic acid (TNBS)-induced colitis and experimental autoimmune encephalitis, respectively. It does not demonstrate anti-inflammatory activity in rats, which is consistent with the absence of SAA in this species. (73, 74). A phase I study of Amilo-5MER was recently completed in healthy young and elderly adult volunteers (Poster Presentation at Pharmacology 2022).

This peptide targets SAA through opposing the hydrophobic and hydrophilic faces of the β-sheet conformation (73). The anti-inflammatory activity of amilo-5MER is thought to derive from its binding with high affinity to pro-inflammatory SAA monomers, thereby interfering with SAA polymerization and aggregation which prevent the monomers from forming hexamers. The hexamers are the activated form of SAA which stimulate monocyte secretion of pro-inflammatory cytokines, particularly IL-6 and Th17 and inhibition of hexamer formation results in modulation of SAA proinflammatory activities.

SAA is easily assayable and various assay methods have been used by research laboratories, including radioimmunoassay, radial immunodiffusion, and enzyme-linked immunoassay (ELISA) (75). Moreover, some assays are used for routine clinical laboratories for a rapid result, fully or partially automated methods such as automated latex agglutination immunoassay and the kinetic photometry of anti-SAA coated latex particles (75). The available assays can detect SAA with different sensitivities, some highly sensitive assays can detect as low as 100 ng/L of SAA. However, a SAA concentration of more than 10 mg/L does not need a highly sensitive essay.

The liver is the main site for SAA expression in which SAA is induced by different cytokines (76). SAA mRNA and protein was expressed in atherosclerotic lesions, smooth cells, monocytes, and macrophages (77–79). In addition, it was shown that SAA mRNA is widely expressed in normal human tissues, mainly the epithelial components of the following tissues; stomach, small, and large intestine, kidney, pancreas, thyroid, tonsil, breast, prostate, lung, skin epidermis, and brain neurons (79). SAA is also expressed in lymphocytes, plasma cells, lymphoid follicles, and endothelial cells lining the blood vessels (77, 79). These findings suggest the extrahepatic production of SAA may be important in situations that do not induce the acute phase response (79). In summary, extrahepatic expression of SAA mRNA and/or SAA protein was reported in different histologically normal tissues, various cells stimulated by inflammatory cytokines, and diseased tissues (80, 81).

## 8. Conclusion

This review supports the hypothesis that SAA is a promising potential target for prevention of hyperinflammatory syndrome associated with COVID-19 and a variety of autoimmune inflammatory diseases. Targeting SAA is considered an early immunological intervention in contrast to drugs that block late-appearing cytokines. In addition, blocking SAA, which has pleotropic effects, is likely to inhibit multiple cytokines involved in the inflammatory cycle and be more effective than blocking a single cytokine. Additional studies linking SAA to the pathophysiology of COVID-19 hyperinflammation and autoimmune conditions to establish the causal relationship and the therapeutic potential of inhibitors targeting SAA activity.

## Author contributions

EA and AL: conception, proposal development, data collection, formal analysis, and manuscript preparation. AF: manuscript preparation. JP: conception, extensive review, input, and editing of manuscript. All authors contributed to the article and approved the submitted version.

## Conflict of interest

The authors declare that the research was conducted in the absence of any commercial or financial relationships that could be construed as a potential conflict of interest.

## Publisher’s note

All claims expressed in this article are solely those of the authors and do not necessarily represent those of their affiliated organizations, or those of the publisher, the editors and the reviewers. Any product that may be evaluated in this article, or claim that may be made by its manufacturer, is not guaranteed or endorsed by the publisher.
